# Abdominal Aortic Aneurysm Type II Endoleaks

**DOI:** 10.4172/2329-9517.1000255

**Published:** 2016-08-20

**Authors:** Mohamed S Kuziez, Luis A Sanchez, Mohamed A Zayed

**Affiliations:** 1 Section of Vascular Surgery, Department of Surgery, Washington University School of Medicine, St. Louis, Missouri, USA; 2 Department of Surgery, Veterans Affairs St. Louis Health Care System, St. Louis, Missouri, USA

**Keywords:** Type II endoleak, Endovascular aneurysm repair, Aortic aneurysm, Arterial embolization

## Abstract

Type II endoleaks occur commonly following endovascular aneurysm repair (EVAR). Although they remain enigmatic, multiples studies have evaluated preoperative risk factors and strategies for prevention of type II endoleaks. Prophylactic treatment of type II endoleaks can include embolization of accessory arteries, as well as complete aneurysmal sac occlusion. Regular post-operative surveillance and screening for type II endoleaks with triple-phase CTA is the standard of care. Aneurysm size and growth rate are factors that predict whether a persistence type II endoleak is hemodynamically significant, and whether it requires treatment with percutaneous trans-lumbar or trans-arterial embolization techniques. Less commonly, type II endoleaks can be repaired using laparoscopic or open surgical ligation of feeder arterial branches. Emerging methods using endovascular aneurysm sac sealing technology may continue to alter the incidence and long-term management strategies of type II endoleaks. Here we review the latest strategies in the treatment of Type II endoleaks following EVAR.

## Introduction

Abdominal aortic aneurysm (AAA) deaths are the 10^th^ leading cause of mortality among men in the United States, and are associated with an annual estimated 150,000 deaths worldwide [1]. Over the past two decades, endovascular aneurysm repair (EVAR) has emerged as the new standard of care in the management of the majority of infrarenal non-ruptured and ruptured AAAs. Advancements in EVAR, and its wide application, have demonstrated compelling trends in decreasing aneurysm related mortality and morbidity [2]. However, re-intervention rates following EVAR are non-negligible, and as a result this has continued to affect the morbidity and cost-effectiveness of this management strategy.

The majority of re-interventions following EVAR are related to peri-operative aortic aneurysm sac endoleaks. It is estimated that one in four patients who undergo EVAR has an endoleak of some kind (Table 1). Particular endoleaks can contribute to aortic aneurysm sac pressurization and continued expansion increasing the risk of subsequent aneurysm rupture. Type I and III endoleaks in particular are widely accepted to indicate inadequate seal of the aortic aneurysm sac by the endoluminal endograft, and are demonstrated to contribute to aneurysm sac expansion. On the other hand, evidence regarding Type II endoleaks is more reserved, and its surveillance, management, and treatment strategies are variable.

## Preoperative Predictors of Type II Endoleak Growth

**Figure 1:**
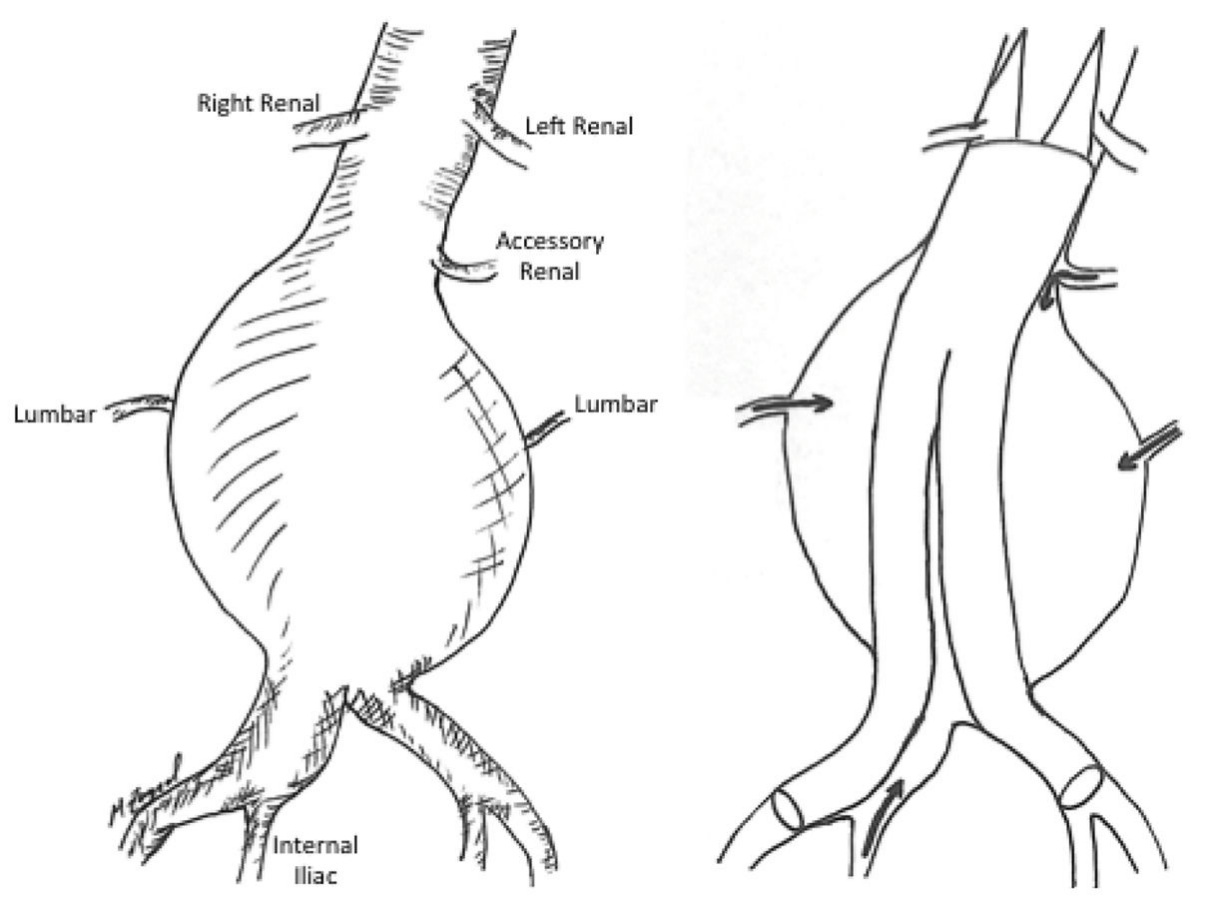
Retrograde blood flow from AAA branches lead to Type II endoleaks. Following EVAR, lumbar, accessory renal, and internal iliac artery branches can lead to retrograde filling of the aortic aneurysm sac leading to Type II endoleaks.

**Figure 2:**
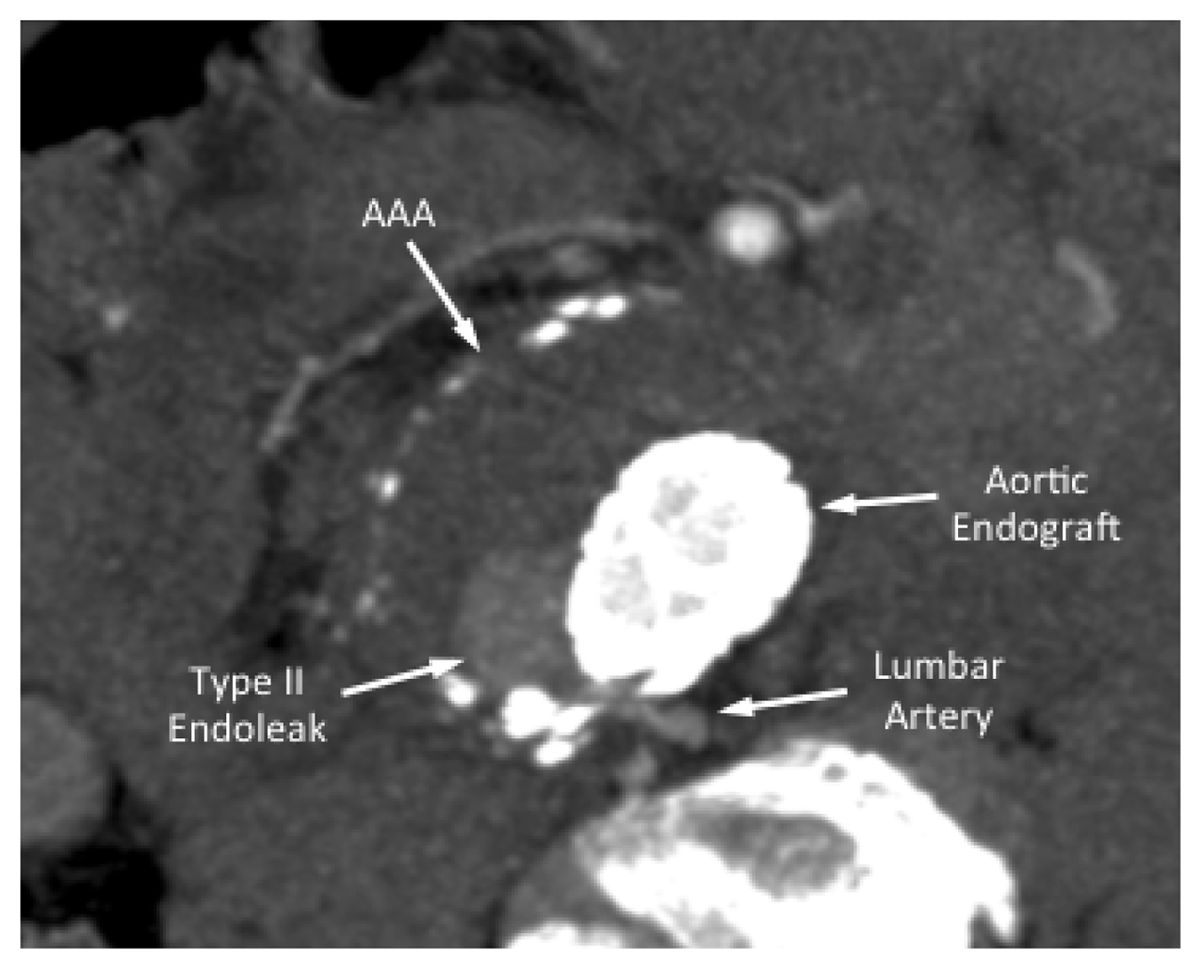
Type II endoleak from a lumbar artery. Following EVAR, follow-up CTA demonstrated delayed focal aortic aneurysm sac filling from a posterior aortic lumbar artery branch.

Type II endoleaks are the most common type of endoleaks that occur following EVAR. They occur from collateral or retrograde filling of the aneurysm sac by one or more of the lumbar, hypogastric, or inferior mesenteric arteries (Figure 1) [3]. Type II endoleaks are further subcategorized as Type IIa, which involve only one vessel (Figure 2), and Type IIb, which involve two or more vessels. In 3595 patients reviewed in the EUROSTAR registry, 9% of patients were found to have a Type II endoleak at least one month following their index EVAR procedure [4]. The subset of patients with detectable Type II endoleaks remained relatively stable over subsequent years (ranging between 6.4–10%; Table 2) [4–13]. Others have also demonstrated variable rates of post-EVAR Type II endoleaks with some studies reporting rates as high as 29%, and others as low as 6.3% [6,14]. In our current practice, it is estimated that approximately 6–15% of patients treated with EVAR develop Type II endoleaks [15,16].

**Figure 3:**
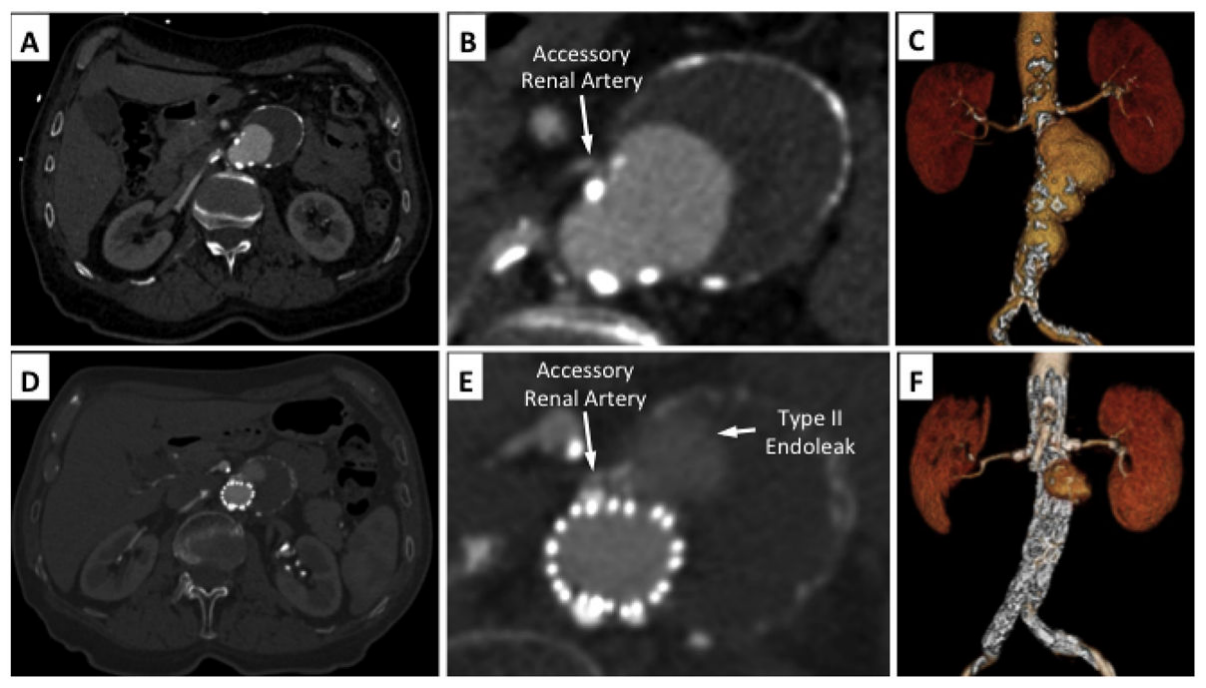
Type II endoleak from an accessory renal artery. (A–C) Preoperative CTA demonstrated a fusiform juxta-renal AAA. Cross-sectional inspection of CTA demonstrated a small accessory left renal artery. (D–F) Postoperative CTA demonstrated a well-positioned fenestrated aortic endograft with patent renal artery stents. A Type II endoleak is visualized directly adjacent to the accessory renal artery from delayed retrograde filling.

Several studies have attempted to identify patient risk factors for development of peri-operative Type II endoleaks. Arko et al. suggested that the number of patent lumbar arteries, or patent inferior mesenteric artery had a significant and strong correlation with both transient and persistent aortic sac Type II endoleaks [17]. Similarly, others have reported that aortic sac diameter and patent accessory renal arteries can lead to persistent aortic sac endoleaks (Figure 3) [5]. In these instances, it is presumed that multiple patent branches emerging from a large aortic aneurysm sac can support antegrade flow from one branch and retrograde flow into another branch. This sustained arterial ‘jet-like’ flow through the aneurysm sac, from one branch to another, can lead to continued aortic sac pressurization and potential destabilization of the aortic endograft repair [5].

Beyond the proposed anatomical risk factors, it is also reported that patient medical morbidities can also influence the incidence of post-EVAR Type II endoleaks. For instance, patients with advanced age, COPD, and systemic inflammatory diseases are reported in some series to have a higher incidence of Type II endoleaks [5,8,18]. However, Type II error is a common limitation in the majority of the studies since aortic anatomy and AAA patient physiology is highly variable. Accordingly, the majority of patients evaluated for EVAR are rarely screened for the procedure solely on their potential risk of developing a post-EVAR Type II endoleak. On the other hand, Society for Vascular Surgery (SVS) guidelines recommends appropriate preoperative planning to prevent other endoleaks such as Type I and III endoleaks [19].

## Intraoperative Predictors of Type II Endoleak Development

The majority of Type II endoleaks result from specific patient anatomical features. However, in unique instances, intraoperative variables may also influence whether a Type II endoleak occurs in the postoperative period. Ward et al. demonstrated that prophylactic embolization of a patent inferior mesenteric artery and/or lumbar arteries decreases the risk of a large persistent Type II endoleak [20]. On the other hand, in repairs that involve distal endograft fixation in the external iliac artery, an inadequate embolization and/or plugging of an internal iliac artery can lead to a persistent retrograde Type II endoleak from the branch vessel and into the aortic sac-particularly if the ipsilateral common and internal iliac arteries are also aneurysmal.

Recent studies demonstrate a strong correlation between how brisk and persistent a Type II endoleak is visualized following aortic endograft deployment. Completion angiograms after EVAR that demonstrated an aortic sac Type II endoleak that persisted for >6 seconds were more likely to be persistent on subsequent postoperative angiographic evaluations (sensitivity 80%, specificity 95%) [21]. It is our practice, that following EVAR a completion angiogram is performed in different obliquities to help determine whether a large Type II endoleak exists [15]. Prescience of these ominous features prompts us to council the patient on the need for continued postoperative surveillance.

Additionally, some investigations have ventured to explore whether particular aortic endografts have differential rates of Type II endoleaks [8,10]. However, the majority of these studies are limited by the size and variability in anatomy of the study participants. It is widely accepted that resultant Type II endoleaks are unlikely a function of the aortic endograft used for the repair, but rather are mainly influenced by the patient’s preoperative aortic and iliac anatomy.

## Postoperative Predictors of Type II Endoleak Development

A large number of Type II endoleaks spontaneously resolve within the first three years following EVAR [4,10]. Some investigators have tried to determine whether specific patient co-morbidities and/or risk factors correlate with incidence of persistent Type II endoleaks. In one study, patients who were cigarette smokers and/or had COPD were less likely to have persistent Type II endoleaks (Table 2) [8]. The authors speculated that perhaps increased hematocrit levels in patients with history of smoking and/or COPD reduces arterial collateral flow within the aneurysm sac, and help with Type II endoleak resolution. Another study separately identified the incidence of coronary artery disease and cancer to be inversely correlated with persistent Type II endoleaks [11].

Some have investigated whether systemic anticoagulation or antiplatelet therapy can influence the incidence and resolution of Type II endoleaks. In a review of 219 patients, use of Coumadin or dual antiplatelet therapy was found to positively correlate with persistent Type II endoleaks [22]. However, the increased incidence of Type II endoleaks did not correlate with an increased need for re-intervention.

## Postoperative Diagnosis and Management

It is customary that at the conclusion of an EVAR procedure a completion digital subtraction angiogram is performed to confirm proper deployment of the aortic endograft, and determine adequate sealing of the aortic aneurysm sac. A delayed phase on this angiogram helps in the detection of Type II endoleaks, and serves as a good baseline reference for subsequent postoperative angiographic studies.

SVS guidelines for management of AAA discuss that 1 month following EVAR a CT angiogram (CTA) can be performed to evaluate the adequacy of the aneurysm repair. Currently, for this purpose a three-phase CTA is considered the “gold-standard” in the diagnosis and confirmation of whether a Type II endoleak is present following EVAR [19]. Large Type II endoleaks, particularly ones that have an inflow and outflow channel, may appear on the early phase of the CTA and become mistaken for a Type I or III endoleak. Small and otherwise non-hemodynamically significant Type II endoleaks may only appear during the delayed phase of the CTA.

The majority of small Type II endoleaks can spontaneously resolve within 6 months from diagnosis [11]. As such, the majority of Type II endoleaks are managed expectantly with regimented surveillance. Following the initial evaluation within 1 month from EVAR, the patient can be evaluated again at 6 or 12 months with repeat aneurysm sac imaging. If a Type II endoleak is visualized on the initial CTA, then a repeat three-phase CTA is preferred at this point to evaluate for endoleak evolution, aneurysm sac diameter changes, and integrity of the endograft repair. In circumstances where Type II endoleaks appear to persist, it is recommended that patients continue to receive regular postoperative surveillance and imaging follow-up [19].

There are specific features on postoperative imaging that would suggest whether a Type II endoleak is hemodynamically significant. For example, a persistent Type II endoleak with concomitant aneurysm sac expansion >5 mm, or a net increase in sac diameter of >10 mm over 6 months, would indicate that the endoleak is likely significant, and requires further evaluation or consideration for repair [23,24]. If the nature of an endoleak is unclear (especially if there is concern that there are also Type I and/or III endoleaks), a dedicated aortogram can be performed in appropriate obliquities and magnifications. Often in these situations one may not definitively rule out whether the Type II endoleak alone is causing sac expansion. Therefore other potential endoleaks (persistent since index EVAR procedure or have developed spontaneously since the procedure) must also be addressed in addition to continued management of the Type II endoleak.

## Treatment of Type II Endoleaks

**Figure 4:**
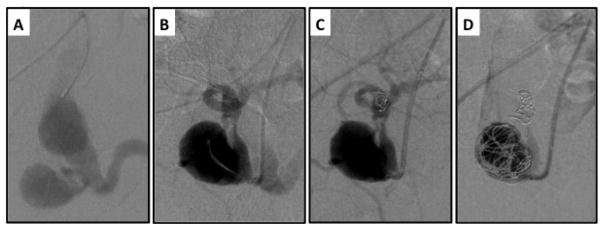
Staged embolization of internal iliac artery prior to EVAR. A) Left iliac artery angiogram demonstrates aneurysms in both the common iliac artery and internal iliac artery. B) The hypogastric artery is catheterized and its distal bifurcation is visualized. C–D) Both the internal iliac artery bifurcation and aneurysm sac are embolized with Nitnol coils to prevent retrograde filling of the common iliac artery aneurysm sac following EVAR with planned distal fixation in the external iliac artery.

Treatment of Type II endoleaks by way of prevention is cited in various reports [19,20,25]. This is theoretically achieved with prophylactic embolization of arterial branches and collaterals that may ultimately lead to a Type II endoleak following EVAR. The most common example of this is pre-EVAR embolization of the internal iliac artery (Figure 4) [19]. This can be performed in a staged fashion, or immediately prior to EVAR during the same operation, and is often a useful adjunct when intending to treat both aortic and common iliac artery aneurysms when an iliac branched device is not indicated or suitable for the iliac artery anatomy.

In a study of 83 patients, Fabre et al. [14] demonstrated that prophylactic embolization of aortic aneurysm sac during EVAR in patients who are at high risk for developing a Type II endoleak, have resultant decrease of postoperative endoleak rate, overall aneurysm sac size, and need for secondary procedures at 6 month follow-up [14]. It is also less commonly proposed that prophylactic embolization can be performed of collateral arteries such as lumbar arteries, accessory renal arteries, or the inferior mesenteric artery [20,23,25,26]. However, it is commonly accepted that the benefit of these prophylactic adjunct interventions does not outweigh the associated procedural risk, cost, and time.

Traditionally, secondary post-EVAR interventions tailored for the treatment of Type II endoleaks are centered on methods used to intentionally embolize the inflow and outflow of collateral arterial branches associated with the aortic aneurysm sac [19,20]. Trans-lumbar intra-aortic sac liquid embolic agents are reported to have high success rates of >80% in embolizing target vessels, but require a unique skill set and a high level of operator expertise [3,27,28]. These include liquid polymer agents such as N-butyl cyanoacrylate (NBCA) glue or ethylene vinyl alcohol (Onyx, Micro Theraputics). Furthermore, the use of specific adjunct imaging modalities, such as CT guided trans-lumbar aortic aneurysm sac cannulation is heavily relied on with this technique [27]. The operator must take extra precautions in following the manufacturer recommendations when preparing the liquid embolic agents, and inject sufficient amounts to facilitate adequate embolization of the endoleak. On the other hand, overzealous use of liquid embolic agent carries increased risk of spinal ischemia, sciatic neuropathy, and/or colonic ischemia [27,29,30].

**Figure 5:**
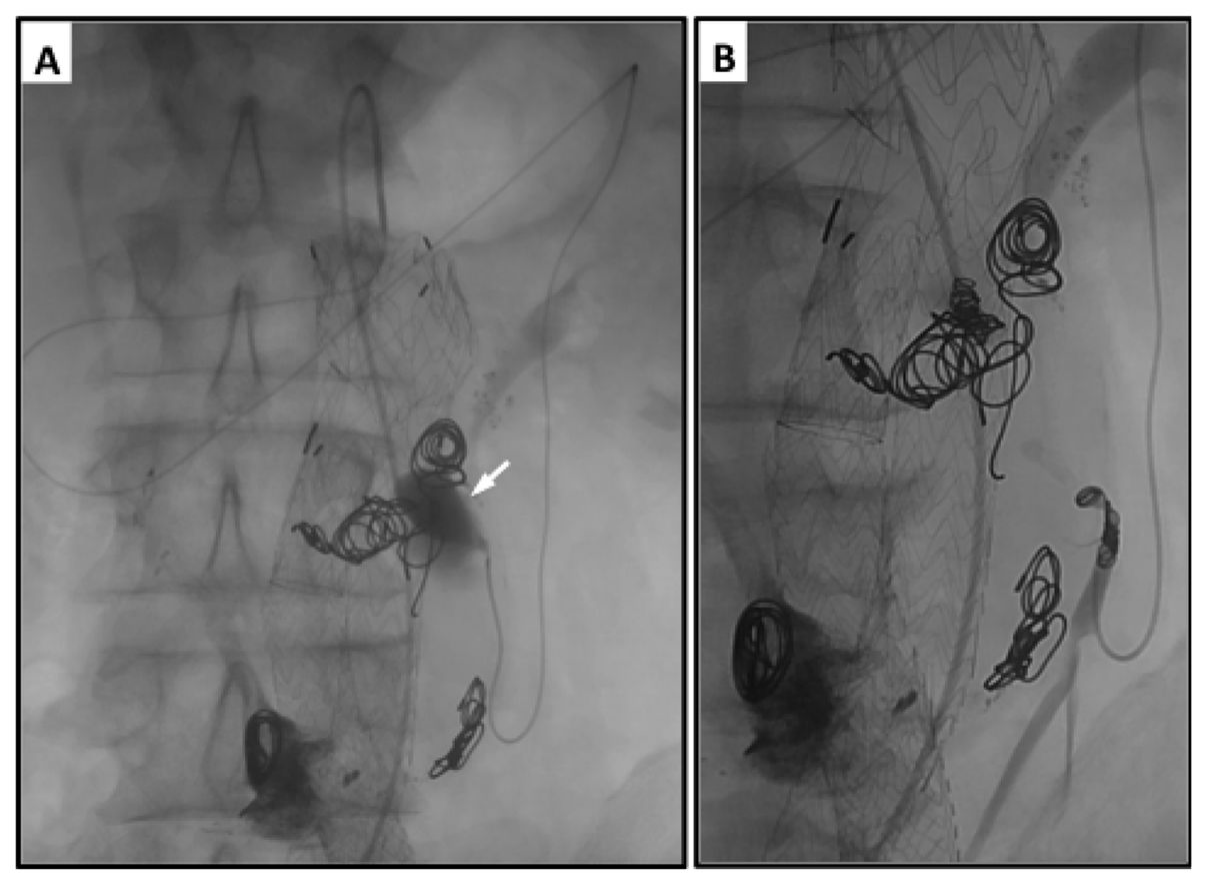
Trans-catheter coil embolization of the inferior mesenteric artery for treatment of aortic aneurysm sac Type II endoleak. A) The inferior mesenteric artery is catheterized using a microcatheter advanced through the superior mesenteric artery and the mesenteric arc of Riolan. Contrast infection through the microcatheter demonstrates type II endoleak from retrograde flow out of the inferior mesenteric artery. B) The inferior mesenteric artery is successfully embolized using Nitnol coils to prevent further filling of the aneurysm sac.

Another conventional method for the treatment and embolization of Type II endoleaks is trans-arterial coil embolization [3]. By way of detailed angiography collateral arterial networks may be selectively catheterized to facilitate advancement of varied coil sizes to vessels suspected of providing either inflow or outflow to the Type II endoleak (Figure 5). Effective disruption of blood flow in these collateral arteries with appropriately sized coils ultimately leads to thrombosis and cessation of the endoleak. However, similar to other embolization strategies, selective catheterization of collateral arcades, and tailored selection and deployment of coils is operator dependent. As such there is reported variable success in achieving sustained Type II endoleak occlusion with these techniques [3,7]. Trans-lumbar and trans-arterial embolization techniques have variable success rates, and are likely contingent on operator expertise and specific patient anatomy [7,27].

Persistent Type II endoleaks can lead to aortic aneurysm sac expansion [31]. Open and even laparascopic, ligation of arterial aneurysm sac branches are described [32]. These techniques can be technically challenging, and may not completely resolve the endoleak despite the more invasive nature of the intervention. Aneurysm sac rupture is rarely described to result from a persistent Type II endoleak, but in these emergent situations, conversion to open AAA repair with graft explanation offers the most definitive method for repair [33].

**Figure 6:**
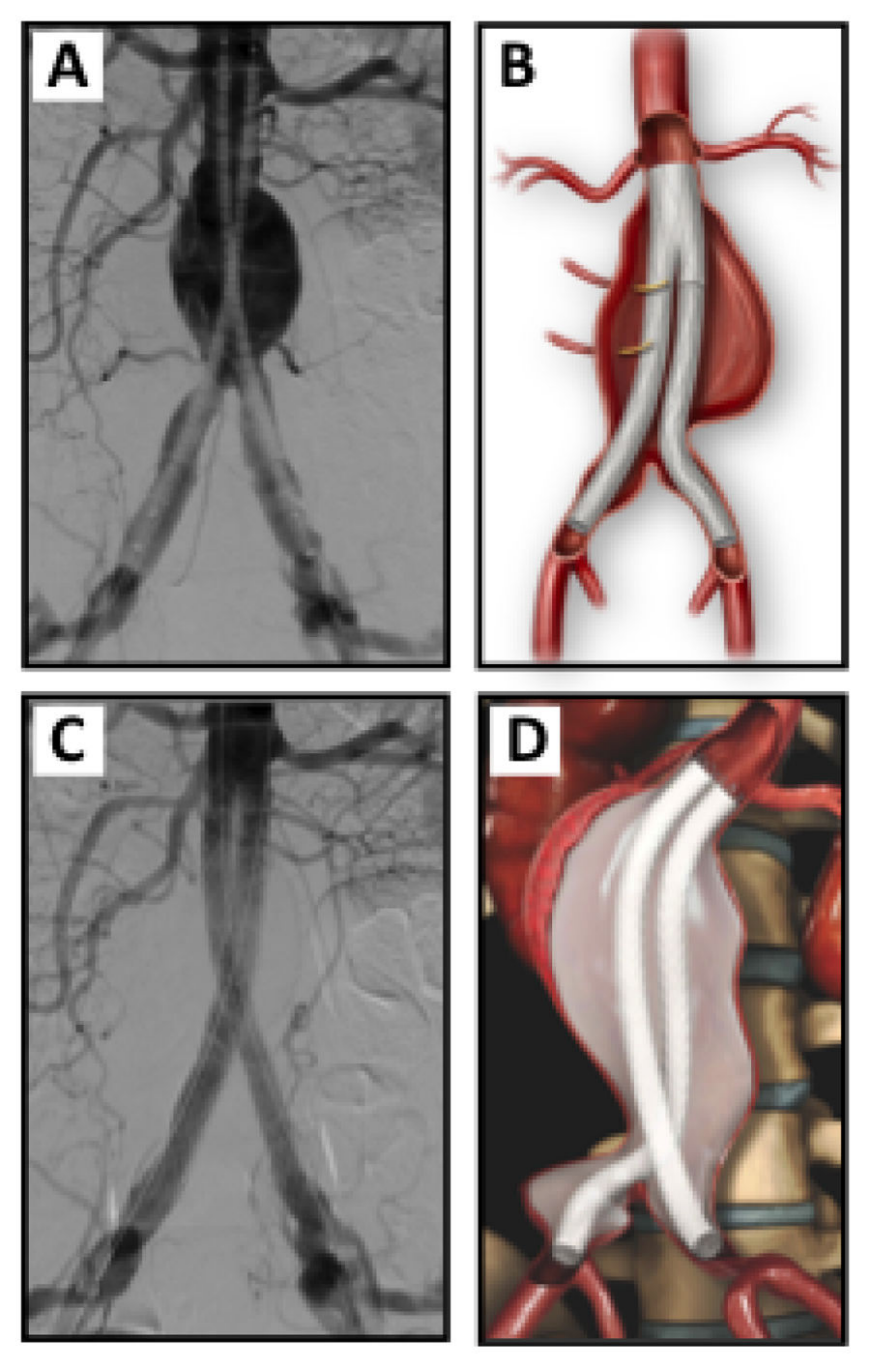
Endovascular aneurysm aneurysm sac sealing with the Endologix Nellix® system. A and B) Endovascular deployment of bilateral aorto-iliac stent grafts that extent from the infrarenal aortic neck to the distal bilateral common iliac arteries. A type II endoleak is visualized via retrograde distal aortic lumbar arteries (A). C and D) Filling of endograft endobag with a biopolymer solution obliterates the aneurysm sac from potential type II endoleaks.

Recently, emerging technology using endovascular aneurysm sealing (EVAS) demonstrated a significant decrease in post-operative Type II endoleak rate, and need for secondary interventions. The Endologix Nellix^®^ EVAS system anatomically bypasses arterial blood flow from the para-renal aorta to the bilateral iliac arteries. In addition, at the time of procedure an endobag surrounding the stent is filled with a biopolymer solution (Figure 6). As the biopolymer solution solidifies it molds to the inner lumen of the aneurysm sac to aid with graft fixation and obliteration of the aneurysm sac space – effectively preventing Type II endoleaks for occurring [33]. In a recent multicenter trial of 171 patients with AAA treated with the Nellix^®^ device, 4 patients (2%) were observed to develop a Type II endoleak at a median follow-up of 5 months. Although the device is not currently FDA-approved in the United States, there is increased anticipation and optimism regarding this technologies’ efficacy in potentially preventing hemodynamically significant Type II aortic endoleaks [34].

## Conclusion

Type II endoleaks following EVAR are common. A large number of these endoleaks are benign in nature and will either spontaneously regress and/or resolve. However, a subset of Type II endoleaks can be hemodynamically significant requiring further surveillance and management. A three-phase CTA is a widely accepted method for detecting and diagnosing Type II endoleaks, but percutaneous angiographic may be employed in selective cases to identify the source of an endoleak and facilitate potential treatment. Minimally invasive percutaneous treatment options include trans-lumbar or trans-arterial embolization of the endoleak and aortic aneurysm sac. However, emerging EVAS technology may provide a novel platform for AAA repair that may further dramatically decrease Type II endoleak rates.

## Figures and Tables

**Table 1:** Overview of type II endoleaks.

Endoleak	Description
Type Ia	Blood flow in the aneurysm sac due to incomplete seal proximally
Type Ib	Blood flow in the aneurysm sac due to incomplete seal distally
Type IIa	Filling of the aneurysm sac due to retrograde branch flow from a single collateral vessel
Type IIb	Filling of the aneurysm sac due to retrograde branch flow from two or more collateral vessels
Type III	Blood flow in the aneurysm sac due to inadequate sealing of overlapping stent grafts
Type IV	Blood flow in the aneurysm sac due to porosity of the graft fabric
Type V	Aneurysm sac expansion with no clear endoleak

**Table 2:** Review of recent studies evaluating rates and complications associated with type II endoleaks following EVAR.

Study	Date	Number of Participants	Mean Length of Follow-up (Months)	Incidence of Type II Endoleaks (%)	In Patients with Type II Endoleaks				
					Aneurysm Sac Enlargement (%)	Resolution (%)	Rupture (%)	Required Re-Intervention (%)	Open Conversion (%)
Van Marrewijk et al. [4]	2004	3595	36	9	19	-	<0.1	22	-
Steinmetz et al. [12]	2004	486	22	18.5	1	62.2	0	1	-
Tolia et al. [13]	2005	83	30	19	0	62.5	0	20	-
Silverberg et al. [11]	2006	956	22	16	20	75	0	12	0
Sheehan et al. [10]	2006	1909	36	16.3	-	37.3	-	-	-
Jones et al. [7]	2007	873	32.6	18.8	-	20	-	-	-
Rayt et al. [9]	2009	369	48	6.7	24	20	0	-	0
Abularrage et al. [5]	2010	595	34.8	23	27.5	25	1.5	28.7	-
El Batti et al. [6]	2013	700	31.3	28.9	40.3	54.2	2	14.9	6
Lo et al. [8]	2016	2367	>6	34	45.7	18.6	4.5	18.6	0.8
